# Quantification of the Surface Coverage of Gold Nanoparticles with Mercaptosulfonates Using Isothermal Titration Calorimetry (ITC)

**DOI:** 10.1021/acs.jpcb.4c03365

**Published:** 2024-10-29

**Authors:** Emilia Tomaszewska, Artur Stępniak, Dominika Wróbel, Katarzyna Bednarczyk, Jan Maly, Małgorzata Krzyżowska, Grzegorz Celichowski, Jarosław Grobelny, Katarzyna Ranoszek-Soliwoda

**Affiliations:** a University of Lodz, Faculty of Chemistry, Department of Materials Technology and Chemistry, Pomorska 163, Lodz 90-236, Poland; b University of Lodz, Faculty of Chemistry, Department of Physical Chemistry, Sub-Department of Biophysical Chemistry, Pomorska 163, Lodz 90-236, Poland; c Centre for Biomaterials and Biotechnology, Faculty of Science, University of Jan Evangelista Purkyně in Ústí nad Labem, Ustí nad Labem 400 96, Czech Republic; d Military Institute of Hygiene and Epidemiology, Laboratory of Nanobiology and Biomaterials, Kozielska 4 St., Warsaw 01-063, Poland

## Abstract

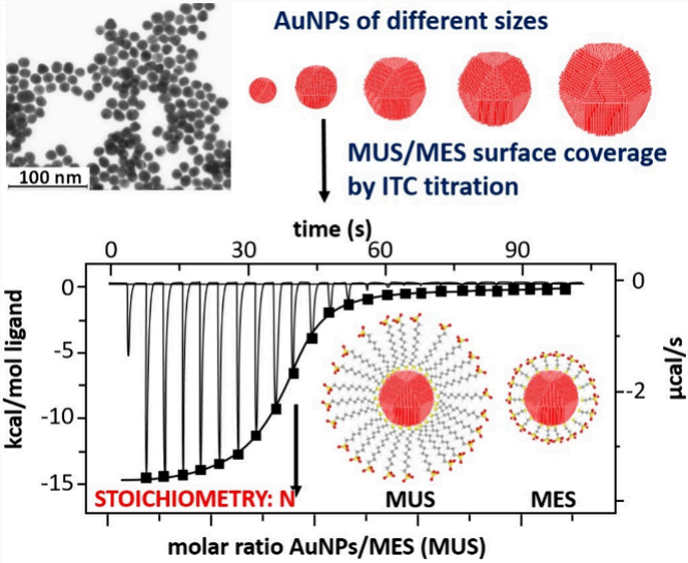

This manuscript presents a comprehensive study on the quantification of modifier molecules adsorbed on gold nanoparticles (AuNPs) using two complementary techniques Ellman’s method (UV–vis spectroscopy) and isothermal titration calorimetry (ITC). In this paper, we compare the feasibility of using the ITC technique and Ellman’s method to study the interactions of mercaptosulfonate compounds (sodium mercaptoethanesulfonate, MES, and sodium mercaptoundecanesulfonate, MUS) with the surface of AuNPs of various sizes. The thermodynamic functions of the attachment of mercaptosulfonates to AuNPs were determined, revealing a linear relationship between the number of adsorbed molecules and the surface area of the nanoparticles. The amount of MES and MUS determined by Ellman’s method (7 and 11 molecules per square nm, respectively) is more than twice that measured by the ITC technique (3 and 4 molecules per square nm, respectively). The slight differences in the adsorption of MES and MUS on the gold surface are due to differences in the carbon chain length of the ligand molecules. In the case of MES, the formation of the Au–S bond is the dominant stage of the adsorption process, whereas for MUS, the ordering process and self-assembly of molecules on the gold surface are dominant.

## Introduction

In recent years, extensive research have been carried out on the use of metallic nanoparticles (MeNPs) in biomedical applications, e.g., as active elements in biomedical sensors,^1,2^ as antibacterial agents, or as drug delivery systems.^3−5^ The use of nanoparticles (NPs) in biomedical applications requires the NPs to undergo precise characterization, in terms of the nanomaterial morphology (size, size distribution, shape) as well as the nanomaterial surface chemistry. The biomedical use of NPs requires the appropriate functionalization of the NP surface for two main reasons: (i) to provide the NP colloidal stability in the biomedical environment, and (ii) to impart a new properties and biological activity to NPs.^6^

Nowadays, NPs are becoming more widely used as antiviral agents.^7,8^ The pathogens constantly mutate and become resistant to the available preparations; hence, an interesting alternative to classical virucidal agents become functionalized MeNPs, especially silver nanoparticles (AgNPs). Scientific reports proved that AgNPs exhibit high efficacy against a diverse range of infections, including herpes simplex virus (HSV-1 and HSV-2),^9^ adenovirus,^10^ dengue,^11^ influenza,^12^ SARS-CoV-2,^13^ and HIV.^14^ Moreover, these studies also indicate that both NP type and the functional compounds present on the NP surface play an equally important role in the final properties of functional NPs. In our previous work, we proved that ligands adsorbed on the NP surface may engage in various interactions with pathogens.^9^ We also demonstrated that the most promising antiviral activity exhibit AgNPs and AuNPs modified with polyphenols^9,15,16^ and sulfonates that mimic the polysaccharide heparin, which plays a crucial role in viral binding to host cells.^17,18^

The use of functional MeNPs in virucidal treatments allows us to obtain additional unique effects. The antiviral effects of MeNPs extend beyond the treatment of active infections, but may also contribute to prevent disease recurrence by stimulating the immune system.^19^ We demonstrated that alterations in the molecules adsorbed on the NPs’ surface can modulate the generation of both cellular and humoral immune responses. MeNPs functionalized with heparin-like compounds are used primarily in the treatment of existing active infections. In contrast, NPs modified with polyphenols act as immunostimulants, thereby aiding in the prevention of disease recurrence. Cagno and co-workers investigated the antiviral properties of NPs fully coated with mercaptosulfonate compounds (specifically sodium mercaptoethanosulfonate—MES and sodium mercaptoundecanesulfonate—MUS).^17^ In this work, the coverage of NPs with the functional ligand was not determined; hence, this methodology did not allow the use of colloids directly for biological research but required the use of a purification procedure before biological studies (to remove the unbound modifier molecules). If the maximum ligand coverage of NPs was known, a specific amount of modifier could be used for modification, and no purification procedure would be necessary (a process that is challenging and may be unfeasible, particularly for small NPs). Moreover, the determination of the surface coverage of NPs with ligand allows the preparation of multifunctional particles, which may contain more than only one biologically active compound but, for example, two functional compounds adsorbed on one NP, e.g., polyphenol and mercaptosulfone compound. However, the main challenge in preparation of multifunctional NPs is the precise identification and quantification of modifier molecules attached to the NPs’ surface. First of all, all structures present in the colloidal solution, particularly those adsorbed onto the metal surface, must be determined. Second, a simple, reproducible, and highly sensitive analytical protocol is needed to determine the stoichiometry of the reaction between MeNPs and the ligand compounds. The determination of the type and quantity of molecules adsorbed on the NPs’ surface presents significant challenges. This process is complex and expensive and requires the integration of multiple advanced characterization techniques. Moreover, certain procedures may result in a loss of colloidal stability of NPs.

Considering the measurement methodology, which can significantly influence the results, the analysis of NP surface coverage can be conducted using several approaches. Most of the available analytical techniques offer indirect quantitative determination of ligands: nuclear magnetic resonance spectroscopy (NMR),^20,21^ Fourier transform infrared spectroscopy (FTIR),^20,22^ dynamic light scattering (DLS) coupled with zeta potential measurements,^23−25^ transmission electron microscopy (TEM),^25−27^ X-ray photoelectron spectroscopy (XPS),^25−27^ thermogravimetric analysis (TGA),^21,28^ and UV–visible spectroscopy.^23,29^ All of the listed techniques do not allow the direct determination of the number of molecules adsorbed on the NP surface, e.g., FTIR spectroscopy reveals shifts in characteristic bands of functional groups present in adsorbed compounds; TEM imaging demonstrates increased interparticle distances and altered NP organization, and zeta potential measurements indicate changes in surface charge due to the adsorption of additional molecules. Moreover, the techniques listed here predominantly yield qualitative rather than quantitative data.

The presented limitation of the techniques mentioned above indicates the need to use other more accurate quantitative methods in the analysis of surface modification. Molecular modeling facilitates the quantitative estimation of adsorbed molecules on the NP surface, based on the predetermined modifier dimensions and their hypothesized arrangement on the metal surface.^26,30^ However, these theoretical predictions require experimental validation. High-performance liquid chromatography (HPLC) offers a quantitative approach to determine the number of molecules adsorbed on the NPs’ surface through two distinct multistep protocols. The first one is the postmodification analysis, which includes the following steps: (i) modification of NPs with an excess of ligand; (ii) separation of modified NPs via centrifugation; (iii) quantification of the concentration of unbound ligand molecules in the supernatant by HPLC; (iv) calculation of the surface coverage from the difference in ligand concentration before and after NP modification.^31,32^ This method provides an indirect but quantitative measurement of surface modification, bridging the gap between theoretical predictions and experimental results. The second method relies on the direct quantification of molecules adsorbed on the NPs’ surface. This approach comprises the following steps: (i) the removal of unbound excess of modifier from the colloid, (ii) dissolution of purified NPs with adsorbed modifier, ensuring the modifier’s structure remains unchanged (e.g., by dissolving gold in KCN), and (iii) determination of the released compound concentration.^33^

An alternative technique for thiol quantification involves a colorimetric assay using Ellman’s reagent.^34,35^ This method, as those described above, allows the determination of the surface coverage via two different protocols: (a) quantification of unbound thiols in the supernatant after NP modification and (b) separation or direct measurement of thiols released upon NP dissolution. These methodologies provide complementary approaches to quantify surface modification, each with its own set of advantages and limitations. In both cases, the modifier concentration is determined through UV–vis spectrophotometry, measuring the absorbance of the complex formed by the reaction of thiol with 5,5′-dithiobis(2-nitrobenzoic acid) (DTNB). Additional techniques for quantification of adsorbed molecules on the NP surface include capillary and gel electrophoresis. These methods allow the analysis without the desorption process, although it is mainly used for detection of protein modifications.^36^ Although there are several indirect methods for determination of the NP surface coverage, they often have some limitations, e.g., the lack of quantitative precision and reliance on multistep procedures, leading to possible measurement errors. These constraints underscore the need for other direct and accurate quantification methods in NP surface modification analysis. TGA offers a direct approach for quantification of the total modifier content on colloidal MeNPs.^37^ While TGA provides a relatively straightforward method for coverage quantification, it also has some limitations, which are mainly the sample size requirements (typically in the range of milligrams or milliliters). Moreover, this technique may not effectively differentiate between varying molecular weight distributions and distinct end-group functionalities. These constraints highlight the need for complementary techniques that can provide more detailed molecular information while maintaining a quantitative accuracy. Isothermal titration calorimetry (ITC) emerges as a promising technique to address those challenges. This method enables the study of molecule–NP surface interactions through the measurement of heat released or absorbed during bond formation. The key advantages of the ITC technique include (i) the possibility of determination of binding affinity and binding stoichiometry of ligand to the NP surface; (ii) the possibility of quantification (determination of the number of molecules adsorbed on a single NP^38^); and (iii) thermodynamic characterization of chemical reaction (stability constant (KD), Gibbs free energy (Δ*G*), and entropy (Δ*S*)). These make ITC a powerful tool for studying the quantitative and thermodynamic aspects of NP surface modification. However, in the case of NPs, the ITC is most commonly used to study the formation of a protein corona on the surface of AuNPs.^39−41^ Nonetheless, there are only a few literature reports presenting the use of this technique for determination of the quantity of adsorbed small-molecule compounds on the surface of MeNPs.^42−44^

Modification of the metal surface can be carried out by at least several functional groups (e.g., amino and thiol groups). Joshi and co-workers described the possibility of using the ITC technique to study the binding of a basic amino acid, lysine, and an acidic amino acid, aspartic acid, to the surface of AuNPs as a function of solution pH.^43^ Results demonstrated that strong bonds form when amino groups are unprotonated and that the attachment of amino acids to the gold surface is an exothermic reaction. The binding isotherms were plotted solely against the total volume of amino acids added to the reaction cell and were used exclusively to identify trends in amino acid binding behavior. Consequently, due to the inability to determine the molar concentration of NPs, the ITC technique was not used to measure the surface coverage. In contrast, Goel and co-workers used ITC to determine the stoichiometry of the reaction between AgNPs and lipoic acid, as well as two peptide sequences.^42^ The modification was achieved by using a thiol group, enabling the covalent binding of the modifying compound to the NP surface. To determine the surface area available for modification, they employed size distribution data obtained from transmission electron microscopy (TEM) and a novel open-access algorithm (NANoPoLC) that calculates the total surface area for samples of varying polydispersities. AgNPs used in their experiments were stabilized with sodium citrate. To replace adsorbed sodium citrate with lipoic acid, concentrations higher than the estimated maximum concentration required to form a monolayer were used. The number of molecules adsorbed on the NPs was determined not only by the available surface area for modification but also by the supramolecular arrangements of the molecules in proximity to the silver. The data obtained from ITC were compared with energy calculations performed using advanced molecular dynamics simulations, enabling the observation of structural behavior of peptides in relation to AgNPs. A literature review reveals that Ravi and co-workers performed a detailed thermodynamic analysis of the adsorption of low-molecular-weight thiol compounds on the surface of AuNPs.^44^ The ITC measurements allow the determination of the thermodynamic parameters of binding and organization of carboxylic acid-terminated alkanethiols with different chain lengths (C2, C3, and C6) on the surface of AuNPs of three different sizes. Analysis of their results indicated that the enthalpy change (Δ*H*) increases linearly with increasing alkyl chain length, as well as with decreasing temperature and AuNP size. Thiol–NP interactions were found to be enthalpy-driven and accompanied by an unfavorable entropic contribution. Moreover, the reaction stoichiometry and number of surface gold atoms per modifier molecule were also determined.

The aim of this study was to determine and to compare the surface coverage of AuNPs with thiol compounds MES and MUS using two different analytical methods: (i) the Ellman’s method and (ii) ITC. A comprehensive characterization (size and shape measurements) was performed for a series of colloids with varying sizes of AuNPs (5, 13, 20, 30, and 40 nm). The analysis of the results allows the key parameters to be calculated, enabling the determination of the degree of surface coverage with MES and MUS in AuNP colloids: (i) the number of NPs; (ii) the surface area available for modification; and (iii) the molar concentration of NPs. Moreover, this study determines and experimentally confirms the quantitative measurements determining the stoichiometry of the AuNP-MES/MUS reaction using the ITC technique. Moreover, based on the ITC measurements, we obtained all thermodynamic parameters of the modification reaction: enthalpy (Δ*H*), entropy (Δ*S*), and Gibbs free energy (Δ*G*), as well as the stability constant (KD) of the formed AuNPs-MES/MUS conjugates. The comparison of the results obtained by both methods allows us to describe the structure of the modifier layer adsorbed on the surface of AuNPs. The determination of the maximum quantity of modifier molecules to fully cover the NPs’ surface allows one to tune the surface chemistry of the NPs to specific requirements. This, in turn, may facilitate the rational design of functional MeNPs to target specific stages of viral infection.

## Methods

### Materials

Gold(III) chloride hydrate (HAuCl_4_·*x*H_2_O, Sigma-Aldrich, ≥49%), sodium borohydride (NaBH_4_, Sigma-Aldrich, ≥96%), sodium citrate tribasic dihydrate (C_6_H_5_Na_3_O_7_·2H_2_O, Sigma-Aldrich, ≥99.0%), sodium 2-mercaptoethanesulfonate (MES, HSCH_2_CH_2_SO_3_Na, Sigma-Aldrich, ≥98.0%), sodium 11-mercaptoundecanesulfonate (MUS, HS-(CH_2_)_11_SO_3_Na, ProChimia, ≥99.0%), 5,5′-dithiobis(2-nitrobenzoic acid) (DTNB, Ellman’s reagent, [−SC_6_H_3_(NO_2_)CO_2_H]_2_, Sigma-Aldrich, ≥98%), sodium acetate (CH_3_COONa, Sigma-Aldrich, ≥99.0%), and Trizma base (2-amino-2-(hydroxymethyl)-1,3-propanediol, NH_2_C(CH_2_OH)_3_, Sigma-Aldrich, ≥99.9%) were used as received. Deionized water obtained from the Deionizer Millipore Simplicity UV system (the specific resistivity of the water was equal to 18.2 MΩ·cm) was used to prepare all solutions.

Gold NPs with sizes of 5 and 13 nm were obtained by the chemical reduction method according to procedures previously described.^31,45^ The synthesis of gold NPs 20, 30, and 40 nm in sizes was based on the seed growth-mediated method.^31^ The procedure was two-stage; in the first stage, gold nuclei of 13 nm were prepared, which were characterized based on STEM measurements. Next, the obtained size was used to calculate the amount of reagents necessary to synthesize 20–40 nm NPs. All syntheses were performed at a final gold concentration of 100 ppm. Briefly, the procedure for NP preparation was as follows.

#### Synthesis of 5 nm AuNPs

Chloroauric acid water solution (28.986 g, 1.786 × 10^–2^ wt %) was added to a flat-bottom flask and vigorously mixed at room temperature. Next, sodium borohydride (1.014 mL, 0.5 wt %) was added, and the solution turned red, which indicates the formation of gold NPs. The colloid was mixed for an additional 1 h.

#### Synthesis of 13 nm AuNPs

The aqueous solution of chloroauric acid (95.4 g, 1.81 × 10^–2^ wt %) was heated under reflux with vigorous stirring. At boiling point, a solution of sodium citrate (4.6 g, 1 wt %) was added to the flask. The colloid changed from yellow to dark red after 2 min. The solution was kept boiling for 15 min and then cooled to room temperature.

#### Synthesis of 20, 30, and 40 nm AuNPs

The synthesis of 20–40 nm AuNPs was as follows: a specific amount of the seed solution, deionized water, and sodium citrate solution were heated to boiling point under reflux. Next, a chloroauric acid aqueous solution was added to the reaction flask through a capillary tube connected to a syringe pump. After the addition of the reagents, the mixture was heated for an additional 15 min, and then the solution was cooled to room temperature. The amount of reagents and synthesis conditions are shown in Table 1. The molar ratios of HAuCl_4_ to sodium citrate were 1:5.5 in all syntheses (20, 30, and 40 nm AuNPs).

**Table 1:** Amount of Reagents Used for the Synthesis of 20–40 nm AuNPs by the Seed Growth-Mediated Method

**NP size [nm]**	**seed colloid [g]**	**H**_**2**_**O [g]**	***C***_**sodium citrate**_**[%]**	**sodium citrate solution [g]**	***C***_**HAuCl4**_**[%]**	**HAuCl**_**4**_**solution [g]**	**rate of addition of HAuCl**_**4**_**[mL/h]**
**20**	20.58	23.71	4.00	0.71	0.045	15.00	15.0
**30**	6.10	37.92	4.00	0.98	0.062	15.00	12.0
**40**	2.57	41.39	4.00	1.04	0.066	15.00	10.0

### Characterization Techniques

#### Dynamic Light Scattering (DLS) and Zeta Potential Measurements

The hydrodynamic diameter and colloidal stability of the obtained gold nanoparticles were characterized using the DLS technique. Measurements were made using a Litesizer 500, Particle Analyzer, Anton Paar. The hydrodynamic diameter and colloidal stability were measured at 25 °C in a quartz and an omega cuvette, respectively. A high-resolution mode was used to analyze the hydrodynamic diameter of the nanoparticles. The zeta potential was analyzed according to the Smoluchowski model. Measurements were taken for the colloids as received, without dilution.

#### UV–Vis Spectroscopy

The maximum absorption band was measured using UV–vis spectroscopy. UV–vis spectra were recorded using a UV–vis spectrophotometer UV-5600 Biosens (METASH) in the range of 200–900 nm. All gold colloid measurements were performed in a quartz microcuvette, while thiol concentration measurements using the Ellman’s method were conducted in disposable fluorimetric cuvettes. The AuNP colloids were diluted to obtain an absorbance below 1. The procedure for determining thiols using Ellman’s method was as follows: initially, calibration curves were prepared in the concentration range of 1–40 ppm independently for MES and MUS solutions. The obtained relationships are presented in Figure S1 in the Supporting Information. The procedure involved mixing 100 μL of a 2 mM DTNB solution in 50 mM sodium acetate, 200 μL of 1 M Tris buffer (pH 8, adjusted with HCl), and 1 mL of H_2_O for MES or 0.3 mL for MUS in a measuring cuvette. A background measurement was performed for the mixture prepared in the same manner. Subsequently, 1 mL of MES or 1.3 mL of MUS at the appropriate concentration was added, and after 5 min, the absorbance was measured at a wavelength of 412 nm. To determine the functionalization of appropriate samples, the procedure was carried out with MES and MUS solutions for AuNPs ranging from 13 to 40 nm in size. The 5 nm AuNP colloid was excluded from the measurements due to the impossibility of completely removing metallic gold from the solution by centrifugation. Functionalization was achieved by incubation of an aqueous modifier solution (0.1%) with the AuNP colloid for 2 h at 25 °C. The amount of MES and MUS used for modification was calculated to correspond to 5, 10, 15, 20, 25, 30, and 35 modifier molecules per 1 nm^2^ of the NP surface. Calculations were performed for colloids with a concentration of 100 ppm, containing spherical NPs with mean particle sizes of 13, 20, 30, and 40 nm, as measured from STEM images. The molar masses used for calculations were 164 and 290 g/mol for MES and MUS, respectively. The degree of surface coverage of AuNPs by MES and MUS was determined by measuring the concentration of unbound thiol after AuNP modification. Following the modification process, the colloid was centrifuged (RCF = 24,000*g*, 15 min) to remove NPs with adsorbed modifier. The amount of unbound thiol in the supernatant was determined using the Ellman’s method. The exact amount of thiol molecules adsorbed onto AuNPs was calculated by comparing the amount of modifier used for modification and the amount remaining unbound. The degree of surface coverage was determined based on the average of three independent measurements.

#### High-Resolution-Scanning Transmission Electron Microscopy (HR-STEM)

The shape, size, and size distribution of gold nanoparticles were determined based on measurements using high-resolution-scanning transmission electron microscopy (HR-STEM) equipped with a STEM II detector (Nova NanoSEM 450, FEI, USA; immersion mode; accelerated voltage = 30 kV). For imaging, a drop of nanoparticle colloid was deposited on a copper grid with a carbon film and allowed to dry. Nanoparticle size histograms were made by measuring 500 nanoparticles for each colloid.

#### ITC

The heat released during the formation of the bond of MES and MUS with the surface of gold NPs of increasing diameter was measured by ITC using a MicroCal PEAQ-ITC calorimeter. Measurements were carried out at a temperature of 298.15 K. The measurement system consisted of the following: the measuring cell with a volume of 280 μL was filled with a colloid of gold NPs, the syringe contained a ligand solution (MES or MUS), and the reference cell contained deionized water. A water solution of gold NPs was titrated with 19 portions of the ligand. The molar concentration of gold NPs necessary to perform calorimetric measurements was determined on the basis of the size and shape determined using HR-STEM and the mass and volume of gold in the colloid. All titration parameters (set concentrations of AuNPs and MES/MUS, as well as injection volumes) are presented in Table 2.

**Table 2:** Detailed Parameters for Performing ITC Measurements

	***C***_**AuNPs**_[mol/L]	***C***_**MES**_[mol/L]	***C***_**MUS**_[mol/L]	**injection volume [μL]**
**5 nm**	1.32 × 10^–7^	1.87 × 10^–4^	3.1 × 10^–4^	2
**13 nm**	7.48 × 10^–9^	1.87 × 10^–4^	3.1 × 10^–4^	2
**20 nm**	2.05 × 10^–9^	1.87 × 10^–4^	3.1 × 10^–4^	2
**30 nm**	6.09 × 10^–10^	1.87 × 10^–4^	1.85 × 10^–4^	1
40 nm	2.57 × 10^–10^	9.35 × 10^–5^	1.85 × 10^–4^	1

The Au-MES/MUS direct interaction effect was calculated by subtracting the ligand dilution effects from the Au-ligand titration effect. Example results for the titration of a 5 nm AuNP colloid, along with the ligand dilution effects performed under the same conditions, are shown in the Supporting Information for MES and MUS in Figure S2A and S2B, respectively. The obtained results were analyzed in the Origin MicroCal 7.0. software, using one type of active site model to mathematically describe the obtained titration curves. Based on the results obtained, the stoichiometry of MES/MUS binding with gold NPs of various sizes (*n*), the enthalpy (Δ*H*), entropy (Δ*S*), the Gibbs free energy (Δ*G*) of the modification reaction, and the stability constant (*K*_D_) of the formed AuNPs-MES/MUS conjugates were calculated.

## Results and Discussion

### Characterization of AuNPs

**Figure 1:**
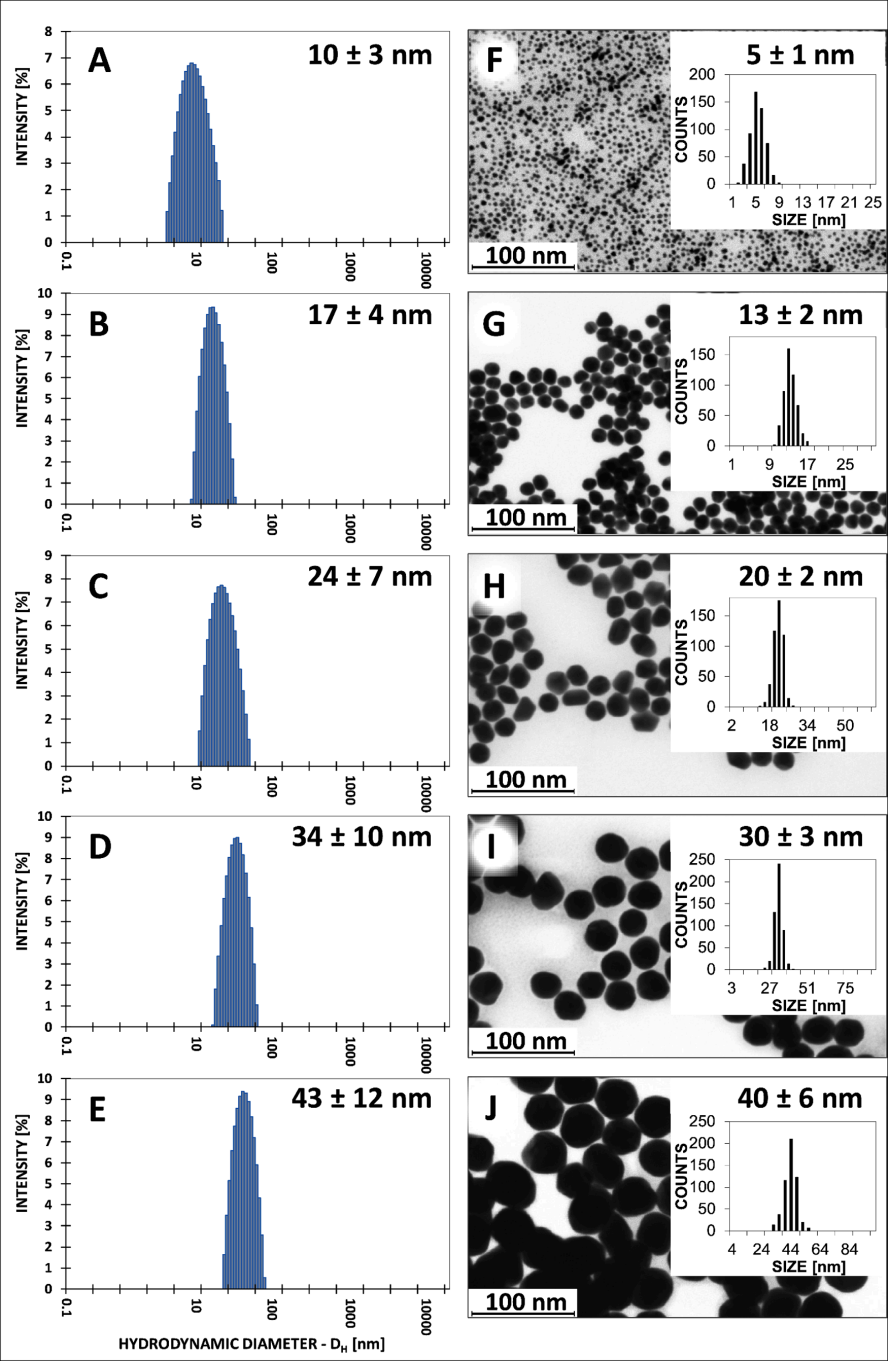
Representative DLS size distribution graphs (A–E of 5, 13, 20, 30, and 40 nm AuNPs) and HR-STEM images with corresponding particle size distributions histograms (F–J) of AuNPs, respectively.

Gold NP colloids were precisely characterized by DLS with zeta potential measurement, UV–vis spectroscopy, and HR-STEM. Figure 1 shows representative DLS size distribution graphs for AuNPs of A—5 nm, B—13 nm, C—20 nm, D—30 nm, and E—40 nm. On the basis of the results achieved, it can be perceived that the colloids obtained are monodisperse; one population is observed in each sample. The STEM images with corresponding particle size distribution histograms are shown in Figure 1 for AuNPs of F, 5 nm; G, 13 nm; H, 20 nm; I, 30 nm; and J, 40 nm, G—13 nm, H—20 nm, I—30 nm, and J—40 nm, respectively. The colloids obtained are homogeneous. The shape of the NPs is uniform, nearly spherical. A 13 nm AuNP colloid was used as seed for the synthesis of particles. No small particles are observed in STEM images of NPs obtained using the seed growth-mediated method (20, 30, and 40 nm), which indicates that the reduction occurred only on the introduced 13 nm seed, and no secondary nucleation occurred.

The results achieved by DLS, STEM, zeta potential, and UV–vis spectroscopy techniques for all colloids obtained are summarized in Table 3. By analyzing the results, it is possible to observe the difference in the sizes of DLS and STEM, which is the result of the specificity of the measurements. In the DLS technique, the hydrodynamic diameter of NPs is measured, i.e., the size of the metal core along with the adsorbed stabilizers. However, in STEM, we get a lower result because only the metallic core is measured. For all samples, the polydispersity index attained by the DLS technique was below 0.2, which confirms the high uniformity of the particles. For all AuNPs, the zeta potential value was obtained in the range of −46 to −37 mV (exact values are presented in Table 3), which, according to the literature, proves the stability of the colloids.^46^ Based on the UV–vis spectroscopy results in Table 3, it can be observed that as the size of the NPs increases, the position of the absorption maximum shifts toward longer wavelengths, which is consistent with the literature.

**Table 3:** Overall Results of Synthesized AuNPs

	***D***_**H**_**[nm]**	**PDI**	***D***_**STEM**_**[nm]**	**zeta potential [mV]**	**λ**_**max**_**[nm]**
**5 nm**	10 ± 3	0.191 ± 0.009	5 ± 1	–40 ± 3	516
**13 nm**	17 ± 4	0.086 ± 0.024	13 ± 2	–37 ± 2	518
**20 nm**	24 ± 8	0.084 ± 0.025	20 ± 2	–37 ± 1	520
**30 nm**	34 ± 10	0.114 ± 0.007	30 ± 3	–42 ± 2	525
40 nm	43 ± 12	0.101 ± 0.009	40 ± 6	–46 ± 2	527

The size obtained based on STEM technique measurements was used to calculate the molar concentration of AuNPs, necessary to perform thermodynamic measurements. The volume of 1 NPs was determined, assuming a uniform spherical shape of the nanoparticles and the established radius of the particle. Then, estimating that the density of gold does not depend on size, the mass of 1 NPs was calculated. The number of nanoparticles in a unit volume of the solution and the molar concentration of nanoparticles were determined based on the mass of gold ions used for the synthesis and the calculated mass of 1 NPs.

### Measurements of Thiol Concentrations Using the Ellman’s Method

**Figure 2:**
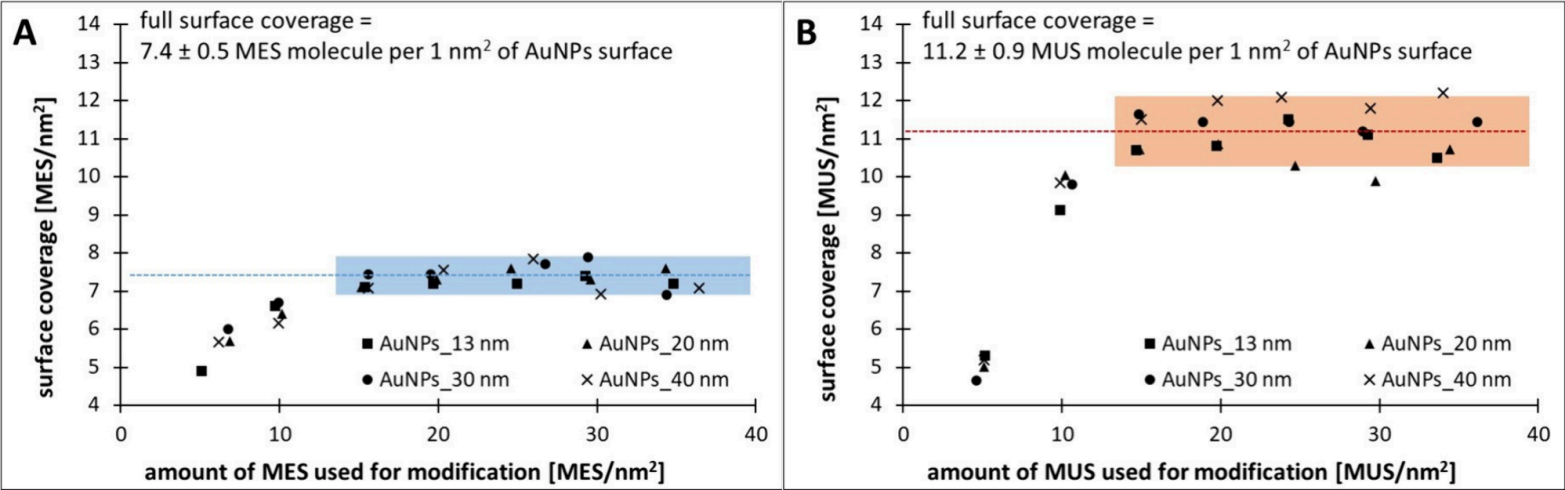
Graphs showing the dependence of surface coverage of AuNPs (13, 20, 30, and 40 nm) by MES (A) and MUS (B) on the amount of thiol used for modification, calculated as the number of MES/MUS molecules per unit of gold surface area (molecules per 1 nm^2^).

The surface coverage of AuNPs with MES and MUS was determined based on measurements of thiol concentration before and after NP modification. As previously described, samples containing AuNPs with varying amounts of thiol were investigated, which were modified with MES and MUS through incubation. The amount of unbound thiols present in the supernatant (obtained from the colloid after centrifugation) was measured using Ellman’s method. Subsequently, graphs presenting the relationship between the amount of MES and MUS used for modification and the amount of thiol molecules present on the AuNP surface were plotted (Figure 2A and 2B for MES and MUS, respectively).

The determined full surface coverage for all AuNP sizes was approximately 7.4 ± 0.5 MES molecules per 1 nm^2^ of AuNP surface (calculated values: 7.2, 7.4, 7.5, and 7.3 MES/nm^2^ for 13, 20, 30, and 40 nm AuNPs, respectively) and 11.2 ± 0.9 MUS molecules per 1 nm^2^ of the AuNP surface (calculated values: 10.9, 10.5, 11.4, and 11.9 MUS/nm^2^ for 13, 20, 30, and 40 nm AuNPs, respectively). The average surface coverage was determined by averaging the values marked in blue for MES and orange for MUS in Figure 2. These results indicate that with increasing modifier concentration up to a certain limit, all MES and MUS molecules adsorb on the gold surface. Beyond this limit, the amount of adsorbed thiol remains constant and only an increase in the concentration of free thiol in the colloid is observed. Therefore, the amount of MES and MUS molecules adsorbed on the AuNP surface can be determined based on the difference between the concentration of thiol used for modification and the measured unbound thiol in the supernatant.

### Thermodynamic Measurements Obtained from the ITC Technique

**Figure 3:**
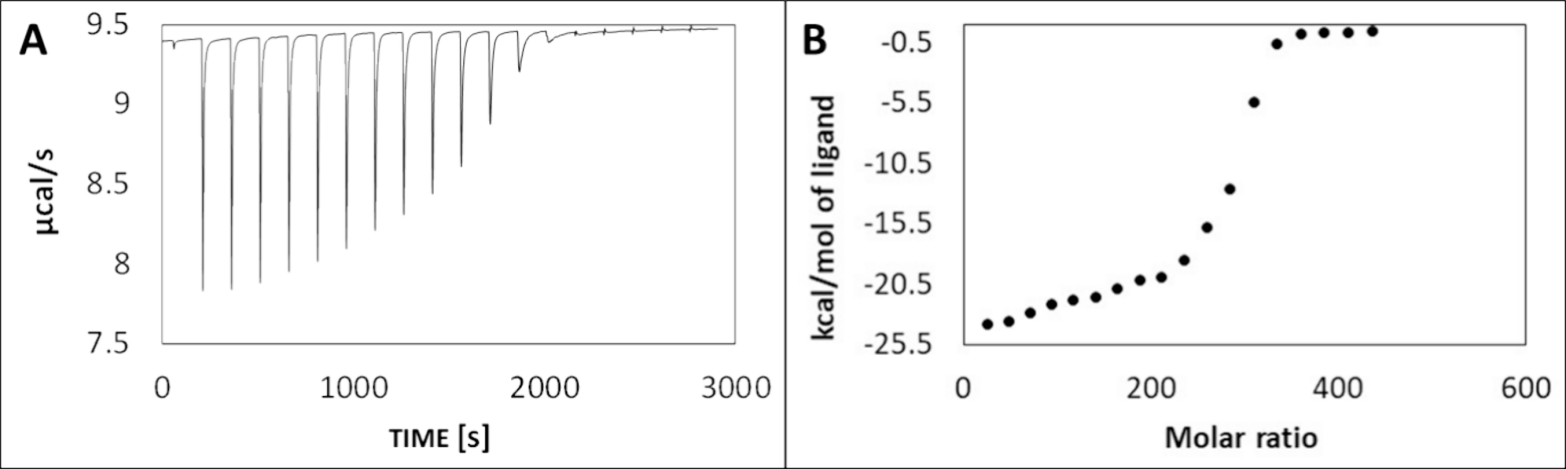
Representative ITC thermogram (A) and corresponding binding isotherm (B) showing the dependence of the reaction enthalpy on the molar ratio of reactants, obtained by integrating the area under each peak of the thermogram, for the titration of a 5 nm AuNP colloid with MUS.

Figure 3 shows an example of a real-time ITC thermogram and the resulting integrated heat data with fitted models. On this basis, various thermodynamic parameters and the number of modifier molecules adsorbed on 1 NP (N) can be determined. The example ITC thermogram shown in Figure 3 can be divided into three characteristic areas, which reveal different stages of formation of the thiol monolayer on the AuNP surface. The first part, before the inflection point, corresponds to the adsorption of MES/MUS on the AuNP surface and the formation of Au–S bonds. All modifier molecules added during the titration step were adsorbed onto the NP surface, and there was no unbound MES/MUS. Then, increasing the amount of modifier molecules led to further adsorption onto AuNPs and the last available area on the AuNP surface. The MES/MUS molecules are organized to saturate the surface (reducing the area under the peak). Finally, after saturation of the entire surface available for modification, we observe the heat of mixing of the thiol with water (no reaction occurs), which corresponds to homogeneous small peaks near the baseline.

**Figure 4:**
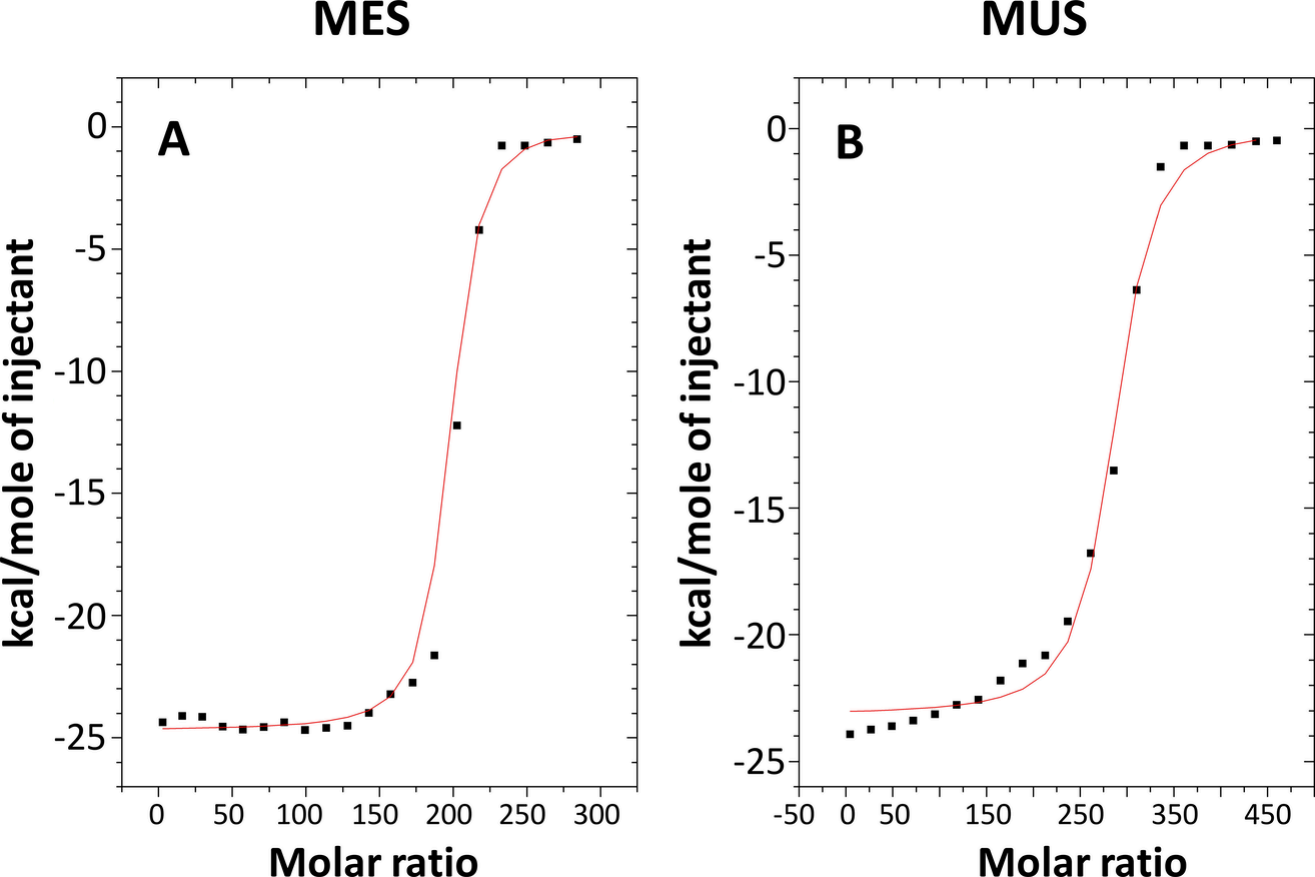
Graphs showing the thermal effects of the direct interaction of nanoparticles with a modifier as a function of the molar ratio for AuNPs with sizes of 5 nm with MES (A) and MUS (B), respectively.

The number of MES and MUS molecules on the AuNPs’ surface was determined on the inflection point of the titration curve. Figure 4 shows the graphs of the thermal effects of the modification process as a function of the molar ratio for AuNP colloids with sizes of 5 nm titrated with MES (Figure 4A) and MUS (Figure 4B), respectively. Graphs for other sets can be found in the Supporting Information in Figure S3. For modifiers with a thiol group, there is one possible way to attach a ligand to a NP, and therefore, a single active site model was used for calculations. The adsorption of alkanethiols on the gold surface consists of the two main stages: (i) the formation of the Au–S bond, which is the fastest stage and responds for about 80–90% of coverage and (ii) the straightening and ordering of alkane chains, which is three to four times slower than the first stage (the formation of noncovalent lateral interactions responsible for ordering of the ligand molecules on the gold surface).^47^ The adsorption of MES and MUS on the gold surface is most probably slightly different, and these differences are caused by differences in the structures of the ligand molecules. In the case of MES, the first stage of adsorption, i.e., the formation of the Au–S bond, is dominant. There is no (or it is very limited) ligand rearrangement on the gold surface due to the too short hydrocarbon chain in the molecule (only two carbon atoms). In the case of the MES ligand, where the molecule consists of 11 carbon atoms, ligand reorganization processes can occur. This may lead to ordering/self-assembly of hydrocarbon chains and consequently lead to higher package of ligand on the gold surface and finally higher surface coverage. Hence, ordering/self-assembly processes are dominant for alkanethiols with long hydrocarbon chains compared to short hydrocarbon chain alkanethiols for which sulfur–gold interface interactions are dominant.^48,49^ This difference in the adsorption process of MES and MUS on the gold surface explains the difference in titration of MUS and MES especially at lower molar ratios (Figure 4 and Figure S3). Some differences were also observed for results obtained for 5 nm AuNPs compared to 13, 20, 30, and 40 nm AuNPs for both MES and MUS ligands. Those differences observed at the initial stage of adsorption may be directly related to the surface chemistry of NPs. Small AuNPs with the size equal to 5 nm are stabilized electrostatically with borates while 13–40 nm AuNPs are stabilized electrostatically with citrates. This may result in the differences in the rate of the first step of the adsorption process (the kinetic of Au–S bond formation), which is influenced by the desorption rates of borate/citrate ions from the gold surface.^47,50^ However, this should only result in a different reaction rate and should not affect the surface coverage, because, as it is known, thiols strongly adsorb on the gold surface and displace adsorbed ions/molecules/contaminations forming repeatable monolayers.^49^

A summary of the thermodynamic parameters obtained from the ITC measurements for the titration of different sizes of AuNPs using the MES and MUS is presented in Table 4. The process of attaching MES and MUS to AuNPs is spontaneous (Δ*G* < 0) and exothermic (Δ*H* < 0), in all tested systems. In all systems studied (mercaptosulfonate compound—gold NPs), negative entropy values (Δ*S* < 0) were obtained, suggesting the appearance of self-organization in solution and an increase in the degree of order of reactants. The total negative entropy value of the reaction suggests that the attachment and ordering of MES and MUS molecules on the surface (Δ*S* < 0) dominate the effect of interaction between AuNPs and electrostatically adsorbed stabilizer (citrate/borate ions) and water molecules, which become detached during the saturation of the gold surface with the thiol (Δ*S* > 0). In addition, in a comparison of the obtained entropy values for MES and MUS, excluding the anomalous behavior of NPs with sizes equal to 5 and 40 nm, two to three times higher entropy values for MUS than for MES can be observed. This probably indicates better ordering of longer MUS molecules on the gold surface, which is also confirmed by the higher number of attached MUS molecules determined by the ITC technique. The adsorption constants are listed in Table 4. The constants for both MES and MUS, regardless of the diameter of the gold NP, have comparable values. Comparing the interactions of MES and MUS with gold NPs, it can be seen that the adsorption constant in all MES interactions with AuNPs is three times higher compared to MUS. These differences may be due to the length of the carbon chain in the ligand molecule.

**Table 4:** Values of Thermodynamic Parameters of AuNP Titration with Sodium Mercaptoethanoslufonate (MES) and Sodium Mercaptoethane Sulfonate (MUS) at a Temperature of 298.15 K

		*n* [thiol/per 1 NP]	***n* [thiol/per 1 nm**^**2**^**]**	***K***_**D**_**[M**^**–1**^**]**	**Δ***H*[kcal/mol]	**Δ***G*[kcal/mol]	**Δ***S*[cal/mol·K]
**MES**	**5 nm**	198 ± 2	2.5	(2.15 ± 0.67) × 10^10^	–24.6 ± 0.4	–14.1	–35.5
	**13 nm**	1840 ± 14	3.5	(2.27 ± 0.64) × 10^10^	–19.0 ± 0.3	–14.1	–16.3
	**20 nm**	5050 ± 53	4.0	(1.74 ± 0.38) × 10^10^	–17.4 ± 0.2	–14.0	–11.3
	**30 nm**	9300 ± 150	3.3	(2.32 ± 0.65) × 10^10^	–18.7 ± 0.2	–14.1	–15.4
	40 nm	16500 ± 350	3.3	(1.13 ± 0.13) × 10^10^	–20.7 ± 0.2	–13.7	–10.4
**MUS**	**5 nm**	285 ± 2	3.6	(6.78 ± 0.14) × 10^9^	–23.2 ± 0.3	–13.4	–32.6
	**13 nm**	2490 + 16	4.7	(7.07 ± 0.83) × 10^9^	–23.1 ± 0.2	–13.4	–32.5
	**20 nm**	5950 ± 71	4.7	(6.29 ± 0.13) × 10^9^	–22.7 ± 0.3	–13.4	–31.0
	**30 nm**	13600 ± 340	4.9	(7.32 ± 0.22) × 10^9^	–22.5 ± 0.3	–13.6	–32.9
	**40 nm**	21600 ± 250	4.3	(4.02 ± 0.66) × 10^9^	–16.4 + 0.3	–13.1	–11.1

**Figure 5:**
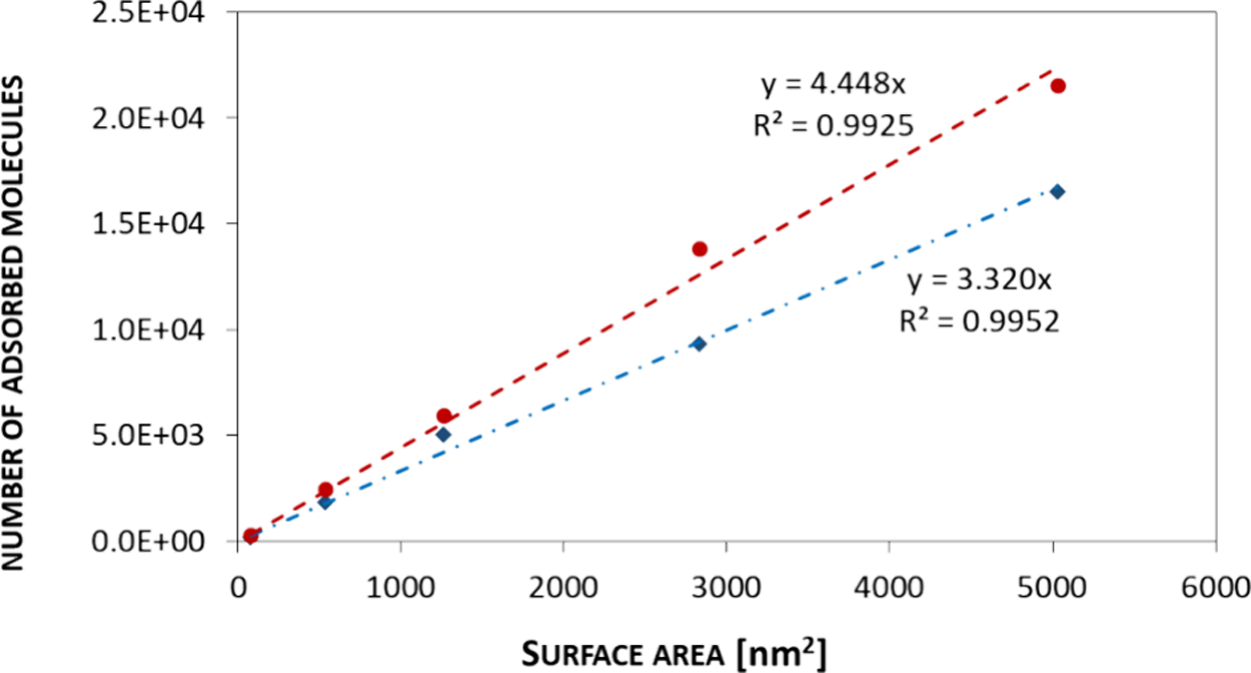
Graph of the dependence of the number of particles adsorbed on the surface of the AuNPs. The dotted line corresponds to the MES titration, and the dashed line corresponds to the MUS titration.

As expected, in the case of the titration of AuNPs using MES and MUS, the amount of adsorbed molecules increases with increasing size (surface area) of the NPs, and the relationship is linear (Figure 5). The linear relationship empowers us to determine the theoretical number of modifier molecules adsorbed on NPs of any size in the 5–40 nm range. The full surface coverage of any size of AuNPs with MES/MUS can be determined on the obtained linear dependence of the adsorbed molecule number on the NP surface area. This can be used in many aspects related to the synthesis and modification of metallic colloids, but it is particularly important in the design of multifunctional NPs. On average, for all sizes of AuNPs, there are 3.3 ± 0.5 MES molecules and 4.4 ± 0.5 MUS molecules per 1 nm^2^. In the case of a shorter MES chain, branched sulfone groups may cause steric hindrance, which prevents the attachment of more molecules, in comparison to a longer MUS chain.

Based on the degree of surface coverage of AuNPs using MES and MUS, determined per number of adsorbed molecules per 1 nm^2^, it is possible to determine how many surface atoms per one modifier molecule there are. In the case of MES adsorption, it is 3.9 ± 0.6 gold surface atoms per 1 modifier molecule, while for MUS, this value is 2.9 ± 0.3. Based on the analysis, it was found that in the case of a modifier with a longer carbon chain (MUS), with full coverage of the NP surface, the access of a smaller number of surface atoms is necessary to adsorb one molecule. The results we obtained are consistent with those previously published in the literature on the subject,^44^ which confirms the appropriate selection of the measurement method and the correct way of analyzing the raw data. The longer chains can increase van der Waals interactions between thiolate molecules, leading to a more compact and stable monolayer. This arrangement may promote the adsorption of more molecules onto the surface. Moreover, in the case of MES, the decrease in the number of adsorbed molecules on the gold surface may be related not only to the limited possibility of interaction of carbon chains but also to the steric hindrance in the form of a large sulfonic group. Although the same group occurs in MUS, its significant separation from the gold surface favors the possibility of adsorption bigger number of molecules per 1 nm^2^.

## Conclusions

The monodispersity of the tested NPs is a crucial factor in understanding the relationship between nanoparticles and a modifier containing a thiol group and in correctly determining the degree of coverage of the metal surface. Therefore, in our work, we presented a chemical synthesis method for obtaining a series of monodisperse gold nanoparticle colloids along with their precise characterization. We emphasize the necessity of using comprehensive nanomaterial imaging techniques to accurately determine the concentration of NPs and the surface area available for modification.

We have provided a comparison of the adsorption studies of two mercaptosulfonic acid compounds (MES and MUS) on the surface of different sizes of AuNPs using two complementary measurement techniques: ITC and Ellman’s method. Each of these techniques yields different information about the adsorption of thiol compounds on the gold surface. The surface coverages determined with ITC and Ellman’s method are comparable. The AuNP surface coverage determined by the ITC technique equals 3 and 4 molecules per nm square of the NP surface for MES and MUS, respectively, and determined by Ellman’s method; 7 and 11 molecules per nm square of NP for MES and MUS, respectively. In the case of ITC, the stoichiometry of the thiol covalently adsorbed on the gold surface was determined based on the adsorption isotherm. Therefore, in this method, the heat of thiol adsorption reaction on the gold surface in direct titration is measured and analyzed. This process requires the proper preparation of starting solutions and a further analysis of the results, while in Ellman’s method, the concentration of unbound thiol is measured. This procedure requires the use of excess thiol for modification. Moreover, the process requires the removal of AuNPs by centrifugation before measurement (plasmon resonance of AuNPs at wavelengths of 520–530 nm). All of these preparation steps can influence the final results obtained with Ellman’s method.

The obtained results and comprehensive thermodynamic analysis of the tested samples revealed that the process of attachment of MES and MUS to the AuNPs’ surface is spontaneous (Δ*G* < 0) and exothermic (Δ*H* < 0). The entropy values of all analyzed connections were negative, indicating the ordering of the system. Moreover, the results indicate that the adsorption of MES and MUS on the gold surface is most probably slightly different, and these differences are caused by differences in the structure of the ligand molecules. In the case of MES (two carbon atoms in the hydrocarbon chain) the formation of the Au–S bond is the dominant stage of the adsorption process, while for MUS (11 carbon atoms in the hydrocarbon chain), the ordering process and molecules self-assembly on the gold surface are dominant.

Based on the ITC measurements, we observed a linear dependence between the number of modifier molecules and the AuNPs’ surface area for both MES and MUS ligands. This relationship can be applied to design multifunctional nanoparticles of any size in the range of 5–50 nm. Knowledge about the number of thiol compound molecules per unit of AuNPs’ surface necessary for their full coverage opens the possibility to plan the synthesis of AuNPs with partial surface coverage. This allows for the introduction of another ligand onto the surfaces of the modified nanoparticles, creating opportunities for the manufacturing of systems with diverse biological activity.

## Supporting Information Available

Ellman’s calibration curves showing the linear dependence of the absorbance on concentration together for MES and MUS; thermograms describing the energetic effects during the titration of a 5 nm AuNP solution with MES and MUS solution with the effects of the thiol dilution; and graphs showing the thermal effects of direct interaction of nanoparticles with a modifier as a function of the molar ratio for AuNPs with sizes of 13, 20, 30, and 40 nm with MES and MUS (PDF)
