# Supplementary material for: Quantification of the Surface Coverage of Gold Nanoparticles with Mercaptosulfonates Using Isothermal Titration Calorimetry (ITC)
Source: J Phys Chem B. 2024 Oct 29;128(44):10904–14. doi: 10.1021/acs.jpcb.4c03365 (PMC11551951; doi:10.1021/acs.jpcb.4c03365)
Supplement: Supplementary file 1 — jp4c03365_si_001.pdf [file jp4c03365_si_001.pdf]

Supporting Information

Quantification of the Surface Coverage of Gold Nanoparticles with Mercaptosulfonates using Isothermal Titration Calorimetry (ITC) and Ellman's method

Emilia Tomaszewska [a*], Artur Stępniak [b], Dominika Wróbel [c], Katarzyna Bednarczyk [a], Jan Maly [c], Małgorzata Krzyżowska [d], Grzegorz Celichowski [a], Jarosław Grobelny [a], Katarzyna Ranoszek-Soliwoda [a]

[a] *University of Lodz, Faculty of Chemistry, Department of Materials Technology and Chemistry, Pomorska 163, 90-236 Lodz, Poland*

[b] *University of Lodz, Faculty of Chemistry, Department Of Physical Chemistry, Sub-Department Of Biophysical Chemistry, Pomorska 163, 90-236 Lodz, Poland*

[c] *Centre for biomaterials and biotechnology, Faculty of Science, University of Jan Evangelista Purkyně in Ústí nad Labem, 400 96 Ustí nad Labem, Czech Republic*

[d] *Military Institute of Hygiene and Epidemiology, Laboratory of Nanobiology and Biomaterials, Kozielska 4 St., 01-063 Warsaw, Poland*

* Email: emilia.tomaszewska@chemia.uni.lodz.pl; Phone: +48 42 6354663

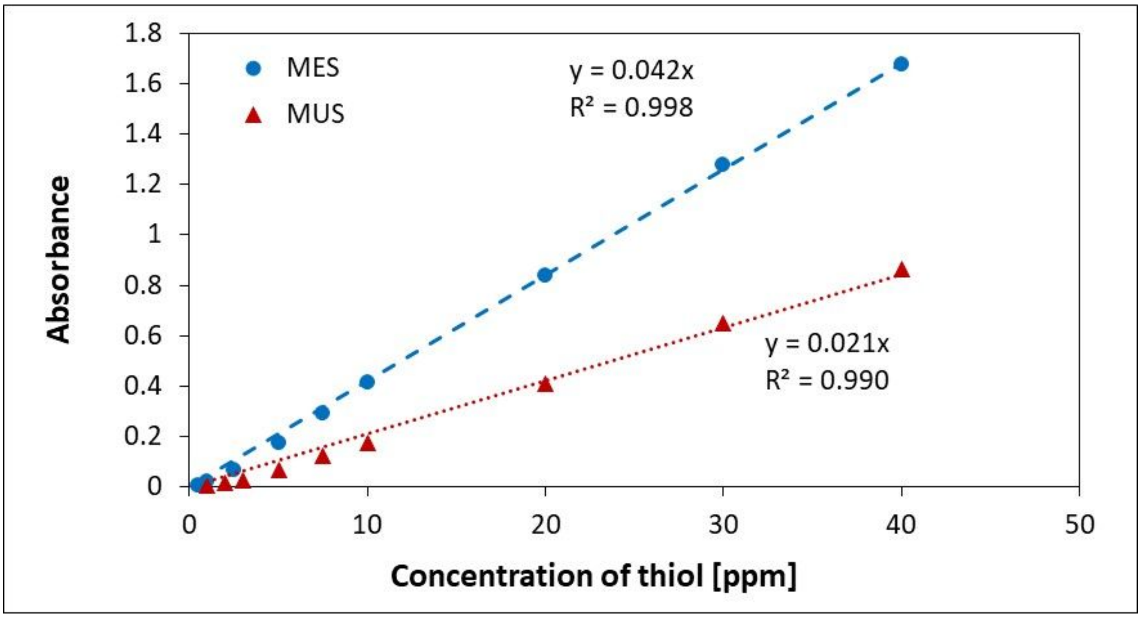

Figure S1. The graph shows Ellman's calibration curves demonstrating the linear dependence of absorbance on concentration for MES (circles) and MUS (triangles). The equation of the line and the coefficient of determination (R^2) are provided for each curve. The slopes of the lines, used to determine the concentration of unbound thiol on the gold surface using the Ellman's method, are 0.042 and 0.021 for MES and MUS, respectively.

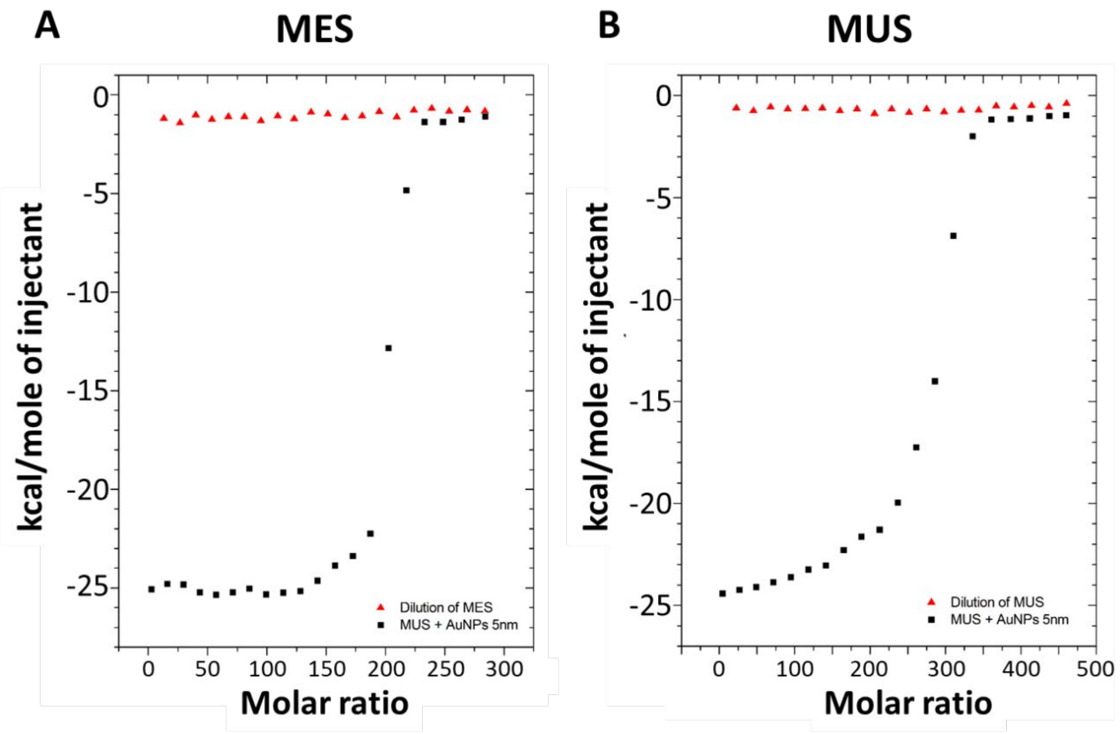

Figure S2. Thermograms describing the energetic effects during the titration of a 5 nm AuNPs solution with MES and MUS solution A and B (squares), respectively, along with the effects of the thiol dilution (triangles).

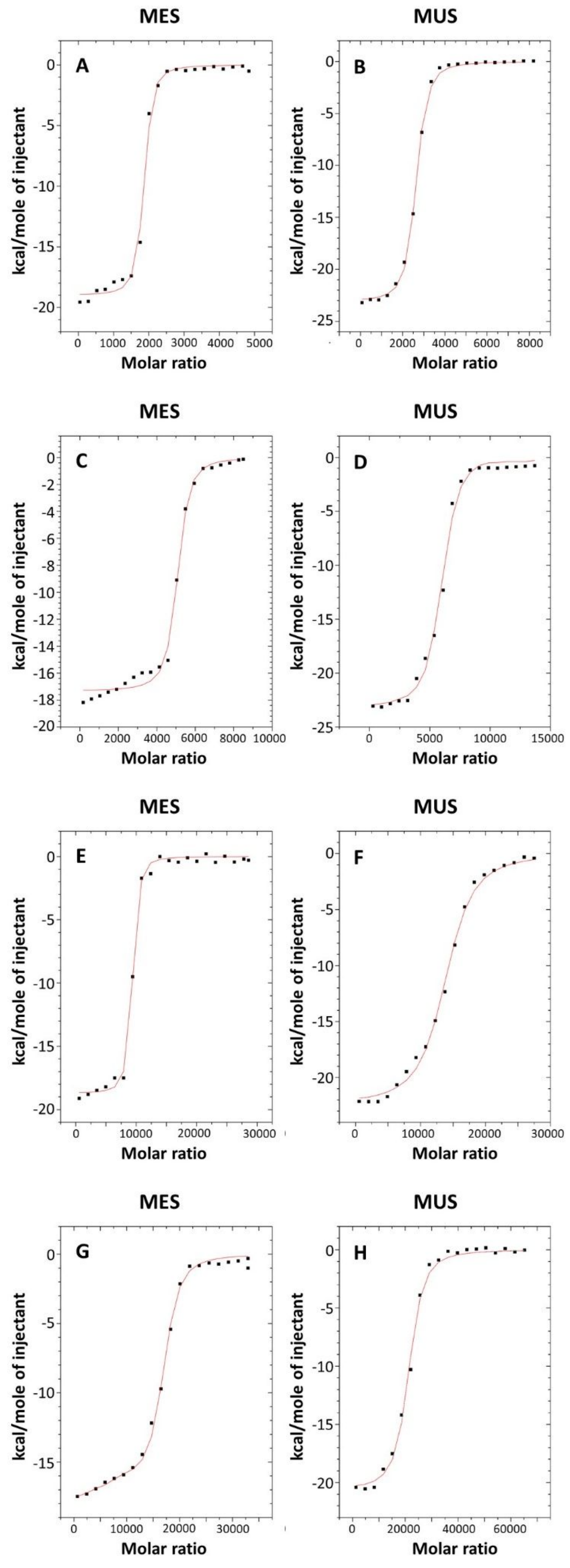

Figure S3. Graphs showing the thermal effects of direct interaction of nanoparticles with a modifier

as a function of the molar ratio for AuNPs with sizes of 13 nm with MES (A) and MUS (B), 20 nm with MES (C) and MUS (D), 30 nm with MES (E) and MUS (F), 40 nm with MES (G) and MUS (H), respectively.
